# RETRACTION: Effect of platelet‐rich plasma combined with negative pressure wound therapy in treating patients with chronic wounds: A meta‐analysis

**DOI:** 10.1111/iwj.70131

**Published:** 2024-11-12

## Abstract

**RETRACTION:** ChenH. , XuT.‐J. , YuH. , ZhuJ.‐L. , LiuY. , YangL.‐P. , “Effect of platelet‐rich plasma combined with negative pressure wound therapy in treating patients with chronic wounds: a meta‐analysis,” International Wound Journal 21, no. 4 (2024): e14758, 10.1111/iwj.14758.38629618 PMC11022301

The above article, published online on 23 April 2024, in Wiley Online Library (http://onlinelibrary.wiley.com/), has been retracted by agreement between the journal Editor in Chief, Professor Keith Harding; and John Wiley & Sons, Ltd. Following an investigation by the publisher, the parties have concluded that this article was accepted solely on the basis of a compromised peer review process. Therefore, the article must be retracted. The authors did not respond to the notice of retraction.

**RETRACTION:** H. Chen , T.‐J. Xu , H. Yu , J.‐L. Zhu , Y. Liu , L.‐P. Yang , “Effect of platelet‐rich plasma combined with negative pressure wound therapy in treating patients with chronic wounds: a meta‐analysis,” International Wound Journal 21, no. 4 (2024): e14758, 10.1111/iwj.14758.38629618 PMC11022301

The above article, published online on 23 April 2024, in Wiley Online Library (http://onlinelibrary.wiley.com/), has been retracted by agreement between the journal Editor in Chief, Professor Keith Harding; and John Wiley & Sons, Ltd. Following an investigation by the publisher, the parties have concluded that this article was accepted solely on the basis of a compromised peer review process. Therefore, the article must be retracted. The authors did not respond to the notice of retraction.

